# Maternal Age and Pregnancy, Childbirth and the Puerperium: Obstetric Results

**DOI:** 10.3390/jcm8050672

**Published:** 2019-05-13

**Authors:** Leticia Molina-García, Manuel Hidalgo-Ruiz, Beatriz Arredondo-López, Silvia Colomino-Ceprián, Miguel Delgado-Rodríguez, Juan Miguel Martínez-Galiano

**Affiliations:** 1 Department of Obstetrics and Gynecology, Jaen Hospital Complex, 23007 Jaen, Spain; letitedi@hotmail.com (L.M.-G.); scolomino@gmail.com (S.C.-C.); 2 Department of Obstetrics and Gynecology, Hospital San Juan de la Cruz, 23400 Ubeda, Spain; manuel9282@gmail.com (M.H.-R.); beayjuanpe@hotmail.com (B.A.-L.); 3 Department of Health Sciences, University of Jaen, 23071 Jaen, Spain; mdelgado@ujaen.es; 4 Consortium for Biomedical Research in Epidemiology and Public Health (CIBERESP), 28029 Madrid, Spain; 5 Department of Nursing, University of Jaen, 23071 Jaen, Spain

**Keywords:** maternal age, obstetric outcomes, pregnancy, labor, puerperium

## Abstract

Delaying maternity is becoming more common, resulting in questions regarding the influence age may have on obstetric results. Therefore, we proposed the objective to determine the association between maternal age and different health variables during pregnancy, childbirth and the puerperium. We conducted an observational study in Spain with primiparous women in which data was collected on sociodemographic, health and obstetric variables. Crude and adjusted mean differences were calculated with their corresponding 95% confidence intervals. The study included a final sample of 373 women. The mean age of women presenting with hypertension during pregnancy was 34.54 years (95% CI: 31.80–37.27) compared with a mean of 30.11 (95% CI: 29.56–30.66) in women that did not (*p* = 0.002). Women who had a eutocic delivery were also younger: 29.17 years (95% CI: 28.48–29.86) compared with 31.90 years (95% CI: 31.05–32.74; *p* < 0.001) for those that had a dystocic delivery. The duration of dilatation was longer in those ≥35 years (*p* = 0.001). In conclusion, an advanced maternal age is associated with a higher incidence of pathology during pregnancy and dystocic labor.

## 1. Introduction

Pregnancy, childbirth and the puerperium constitute a physiologic and natural period in a woman’s life that generally progresses spontaneously and without complications; although complications may arise that affect the health and life of those involved (mother, embryo, fetus, neonate) [1,2].

Since the middle of the 1970s, the delaying of maternity has become an important social phenomenon, due in part to the need and desire to establish better professional preparation, better economic conditions, and work and family stability [3].

The question of female life-expectancy and age of parity has been debated over many years without a consensual conclusion, although it is unanimously accepted that extreme ages (<16 or >35 years) [4] are associated with the development of obstetric complications and increase infant-maternal morbidity and mortality [3,5,6].

Different studies have investigated the impact of maternal age in pregnancy, childbirth and postpartum outcomes [3,5,6,7,8,9,10,11]. A study of historic cohorts conducted in Spain with 1455 women concluded that an advanced maternal age is associated with more gestational pathologies such as gestational diabetes, first trimester metrorrhagia and threatened preterm labor (TPTL), an increased incidence of medical induction of labor, cesarean section rate, perinatal mortality and maternal morbidity [3]. In a cross-sectional study conducted in Cuba with 372 pregnant women, an older age was associated with increased vaginitis, obesity, anemia, pregnancy-induced hypertension, oligohydramnios, dystocic labor with cesarean sections and hyperbilirubinemia [10]. In recent years, the benefits of delaying maternity have also been investigated [12,13,14,15]. In the United Kingdom, research conducted in 2017 with data from three British studies concluded that children born to mothers older than 35 years demonstrated better cognitive abilities than those with younger mothers. In this same study mothers that had children later showed healthier behaviors during pregnancy [13].

However, the delaying of maternity is a common phenomenon world-wide and is becoming more and more frequent, while to date, studies show inconsistent impacts of this phenomenon. Therefore, we aimed to determine the association between maternal age and different variables related to pregnancy, childbirth and the puerperium.

## 2. Materials and Methods

An analytic observational study was conducted including pregnant women who gave birth during 2017 in different hospitals in South Spain. The inclusion criteria were primiparous pregnant women, with a singleton pregnancy and who were not minors (>18 years). Women that had difficulty communicating in Spanish were excluded (language barrier).

Ethics approval was obtained from the Research Ethics Committee of the participating hospitals in this study. Informed consent was obtained from all women participating in this study and the established protocols for the respective health centers were followed for access to data in medical records and to conduct this type of study, with the objective to publish/disseminate the results to the scientific community.

The principal outcome was the appearance of a pregnancy-associated pathology. The sample size was based on the study by Heras Pérez et al. in 2011 [3], in which the incidence of pregnancy-associated conditions in women older than 35 years was 29.2% compared with 15.8% in women 35 years or younger. To assess a difference between those figures with a power of 80% and a statistical significance of 5%, it would be necessary to include 302 women. Taking into account an expected drop-out rate of 15%, a final total of 373 women were recruited. Women were selected consecutively.

### 2.1. Data Collection

Data were collected using a questionnaire which had previously been piloted. The questionnaire was heteroadministered by qualified personnel with knowledge of pregnancy, child birth, and the puerperium, in the immediate puerperium via an interview (two hours after childbirth), and two months after childbirth via a phone call. The majority of the data were obtained through the clinical interview and via the phone call made by a health professional following childbirth; the data were then completed with access to the clinical history and the Pregnancy Health Document.

Data were collected on the sociodemographic variables of the pregnant women, variables related to obstetric antecedents, the current pregnancy, its follow-up and evolution (pathology during pregnancy, number of visits to the emergency room and hospital admissions) and variables related to childbirth (gestational week of childbirth, labor onset, need for medication during childbirth, use of epidural analgesia, duration of labor, amniotic fluid color, cardiotocography (CTG), perceived pain level of the woman during childbirth, type of delivery, type of third stage of labor, perineal lesion and postpartum complications (e.g., fever, hemorrhage or the need for surgical intervention, among others).

To evaluate the perception of pain experienced by women during labor the verbal rating scale (VRS) was used [16].

### 2.2. Data Analysis

Continuous variables were assessed by comparison of means, t-test or analysis of variance. The analysis of covariance was used to estimate adjusted means for potential confounders. For categorical variables, odds ratios (OR) and their 95% confidence intervals (CI) were computed using logistic regression to adjust for confounding. Confounders were considered those variables which were non-intermediate variables and changed the coefficient of the main exposure (maternal age) by more than 10% in multivariate models.

## 3. Results

373 women were included. The mean age of participating women was 30.45 ± 5.63 years. Around 62.2% (232) were married and 98.1% (366) were Spanish. Women with university education made up 37.8% (141) of the study sample and the mean income of 36.7% (137) was between 1000–1999 Euros per month. Around 71% (265) worked during pregnancy and 12.4% (26) had some type of pathology prior to pregnancy. A previous history of miscarriage was reported in 24.9% (93). The pregnancy was planned in 87.4% (326), with 12.3% (46) requiring medical assistance to achieve pregnancy. The mean gestational week in which childbirth occurred was 39.43 ± 1.41, as shown in Table 1 which presents the study population characteristics.

In Table 2 the association between maternal age and different pregnancy, childbirth and puerperium variables are shown. The mean age of women that presented with health problems during pregnancy was 30.66 years (95% CI: 29.89–31.43) compared with 29.90 years (95% CI: 29.13–30.68) in those that did not develop any pathology (*p* = 0.177). A significant difference was observed for hypertension; the women that suffered from hypertension had a mean age of 34.54 years (95% CI: 31.80–37.27) compared with a mean of 30.11 (95% CI: 29.56–30.66) years in women that did not suffer from hypertension (*p* = 0.002). A significant difference was also observed for gestational diabetes, with a mean age of 36.01 years (95% CI: 33.95–38.07) in women that developed gestational diabetes compared with 29.88 years (95% CI: 29.34–30.42) in women that did not have gestational diabetes (*p* < 0.001). In terms of the different childbirth parameters analyzed, the mean age of the women with spontaneous onset of labor was 29.10 years (95% CI: 28.33–29.88) compared with women who required intervention to start labor, who had a mean age of 31.35 years (95% CI: 30.62–32.10; *p* < 0.001). Women who had a eutocic delivery were also younger with a mean age of 29.17 years (95% CI: 28.48–29.86) compared with women who had a dystocic delivery; mean age 31.90 years (95% CI: 31.05–32.74; *p* < 0.001).

Table 3 shows the association between different pregnancy, childbirth and puerperium variables, stratifying for maternal age in 4 categories: <25 years, 25–29 years, 30–34 years, and ≥35 years. Women who were ≥35 years had a positive association with the incidence of gestational diabetes compared with those how were <29 years (a OR = 15.18, 95% CI: 3.03–76.00). A positive association was also observed between women without spontaneous onset of labor and maternal age: Women ≥35 years were more likely to have non-spontaneous labor onset (a OR = 5.87, 95%CI: 2.47–13.96). A dystocic delivery was more frequent in women ≥35 years compared with women ˂25 years (a OR = 6.92, 95% CI: 2.74–17.48). A maternal age ≥35 years was also a risk factor for the presence of stained amniotic fluid compared with women younger than 25 years (a OR = 4.61, 95% CI: 1.42–14.99).

Table 4 shows the existing association between maternal age and pregnancy, labor, delivery and puerperium continuous variables. In Table 4 we observe that younger women, <25 years specifically, are the group that visit the emergency department most frequently during pregnancy, with a mean number of visits of 1.95 ± 0.24 compared with those that are 30–34 years, who least use this service with a mean number of visits of 1.28 ± 0.14 (*p* = 0.087). The duration of dilatation was shorter in the <25-year-old age group, with a mean duration of 290.92 ± 27.87 min, compared with those who were ≥35 years, who had the longest duration with a mean duration of 398.68 ± 19.89 min (*p* = 0.001). The mean duration of the second and third stage of labor did not show significant differences between age groups (*p* > 0.05).

## 4. Discussion

In the present study, an advanced maternal age is associated with a higher incidence of gestational pathology: hypertension and diabetes during pregnancy, a higher incidence of non-spontaneous onset of labor, increased frequency of meconium stained amniotic fluid, non-reassuring CTG recording, longer labor, labor dystocia including cesarean section and non-physiologic third stage of labor, as well as postpartum complications.

In our results, the women that developed hypertension during pregnancy were older than those that did not. Heras Pérez et al. found more frequent hypertension during pregnancy in older women, but this difference did not reach statistical significance [3]. In a retrospective paired cohort, Favilli et al. compared women 40 years and older with a control group of women between 20 and 30 years old and concluded that pregnancy-induced hypertension and pre-eclampsia were similar in both groups [17]. Nevertheless, Bekir Kahveci et al. in a case-control study conducted in Turkey with 957 women stratified in three groups according to age, found an association between maternal age and hypertensive disorders during pregnancy, and concluded that the risk of pregnancy-induced hypertension and pre-eclampsia was significantly lower in women younger than 35 years [18]. Similarly, Tipiani Rodríguez et al., in a case-control study conducted in Lima with 490 women, found an incidence of chronic hypertension of 3.4/1000 in women younger than 34 years, and 21.3/1000 in those between 35 and 44 years [19]; consistent with findings in other studies [6,8,9,10].

In women ≥35 years a positive association with gestational diabetes was found, consistent with results from a large number of studies [3,6,9,17,18,20,21]. Nevertheless, Moya Toneut et al. in a study conducted in Cuba with 372 pregnant women aged 35 years or older, did not observe that gestational diabetes was a pathology predominant in older women [10].

Older women had a higher probability of not starting labor spontaneously, in line with results from Ludford et al. in a study conducted in South Australia using 34,695 perinatal records [22], and those of Prysak et al. using records from three suburban hospitals in the USA [20].

An increased maternal age was associated with longer labor—concretely, a longer period of dilatation was recorded in women ≥35 years—consistent with a retrospective study by Ann Treacy et al. conducted in Dublin with 10737 nulliparous women [23]. However, Sandoval et al. in a case-control study conducted with 324 Peruvian adolescents, concluded that women younger than 20 years have a longer stage of labor and a longer third stage of delivery than those ≥ 20 years, although this did not reach significance and he associated the difference with poorer physical and psychological preparation for maternity [24].

Among intrapartum complications, the presence of meconium stained amniotic fluid constitutes a perinatal risk factor that increases neonatal morbidity and mortality; fundamentally due to perinatal asphyxia (56.1%), respiratory pathology (34%), and digestive pathology (30.5%) [25]. Its incidence in our study was higher in women aged 35 years or older, similar to the results of Moya Toneut et al. [10].

Kim et al. found a higher cesarean rate due to a nonreassuring fetal heart rate increase in mothers over 40 years of age [5]. This was in line with our results, as we also found a greater presence of non-reassuring CTG recording in older mothers.

A dystocic delivery, including both instrumented vaginal delivery and cesarean section, was more frequent in the older age groups—concretely in women ≥35 years—consistent with other studies [3,5,9,10,17,18,23,26]. In fact, Nolasco-Blé et al. in a study of 163 women older than 40 years that gave birth in a hospital in Mexico found a cesarean rate of 71% [27]; well above the recommendations of the World Health Organization (WHO) of a cesarean rate of around 10–15% [11]. In comparison, Bekir Kahveci et al. differentiated between a cesarean delivery and an instrumental delivery, finding a higher rate of cesarean sections in the oldest maternal age group (>40 years), but found no significant differences in rates of instrumental deliveries between groups [18].

In terms of the type of third stage of labor, in our results we observed that spontaneous or physiological third stage of labor was more frequent in younger women. Along the same lines Olortegui Ramos, in a study conducted in Peru with 391 women, concluded that one of the most frequent obstetric complications in older pregnant women was an incomplete delivery [28].

In contrast to our results, Bekir Kahveci et al. associated an advance maternal age with a higher probability of a late preterm birth between 34 and 37 weeks but not a spontaneous premature delivery before 34 weeks [18]. Additionally, in terms of the use of epidural analgesia, Fernández-Guisasola Mascías et al., in a study conducted in Madrid with 3407 women, observed that women that used epidural analgesia were younger than those that did not [29], coinciding with other authors [6]. In contrast, we did not observe this association in our results. Neither did we find an association between maternal age and the rate of episiotomy. However, in a retrospective study conducted in South Spain by Molina Reyes et al. on episiotomy rates in 2560 births, it was found that an episiotomy was more frequently done in nulliparous older women [30]. Balestena-Sánchez et al., in a study conducted in Cuba with 1080 women, highlighted an association between advanced maternal age and postpartum and puerperium complications [31], consistent with our results.

Older women had perineal tears less frequently, consistent with findings by Abril González et al. in a prospective cohort study of 149 women in Columbia in which they identified perineal tear risk factors, noting that a young maternal age—concretely younger than 22 years—was associated with high rates of perineal tears grade II or higher, however this result did not reach significance [32]. In contrast with our results, a study by Sánchez et al. in Lima observed that perineal tears occurred with a higher frequency in women older than 35 years (*p* = 0.05) [33]. They did not establish any association between maternal age and other variables related to pregnancy, birth and the puerperium, such as the number of visits and use of emergency services, hospital admission during pregnancy and the number of days as an inpatient postpartum, preterm delivery and the use of medication during dilatation, and the level of pain perceived by the women during childbirth. Similar to our results, with regards to the woman’s perceived pain level during labor, Chang et al., in his study on demographic and obstetric factors and labor pain in 90 primiparous women that had normal deliveries in a hospital in South Taiwan, also concluded that perceived pain in each stage of labor did not have any significant association with maternal age [34]; consistent with another study [35].

It is possible that maternal age influences in some way the decisions that are made by the health personnel. This may lead to an increase in interventions in the birth process such as non-eutocic deliveries, non-spontaneous onset of labor, etc.

Our study sample is representative of the population. The questionnaire used to collect the data has been previously piloted. The developed questions were clear and understandable for all educative levels, eliminating any information bias. It is not possible to completely eliminate memory bias, however, we believe—a priori—that the influence of this bias on our results were insignificant due to the type of information collected and the short time period at which the questionnaire was administered. A selection bias associated with non-responders is unlikely to have had an influence on the results, as the majority of women selected agreed to participate. Only 13 woman refused participation and there are no indications that this group would have responded differently to those that did participate.

## 5. Conclusions

An advanced maternal age was associated with an increased presence of hypertension during pregnancy, gestational diabetes, non-spontaneous onset of labor, stained amniotic fluid, non-reassuring CTG recording, longer labor, dystocic delivery including cesarean section, non-physiologic third stage of labor and more frequent postpartum complications.

## Figures and Tables

**Table 1:** Characteristics of the study population.

Variable	
Mean age (SD)	30.45(5.63)
Civil status, *n* (%)	
Single	102 (27.3)
Married	232 (62.2)
Defacto relationship	36 (9.7)
Divorced	3 (0.8)
Nationality	
Spanish, *n* (%)	366 (98.1)
Other	7 (1.9)
Education level, *n* (%)	
No education	8 (2.1)
Primary	25 (6.7)
Secondary	105 (28.2)
Upper secondary *	94 (25.2)
University	141 (37.8)
Income, *n* (%)	
<1000 Euros	98 (26.3)
1000–1999 Euros	137 (36.7)
2000–2999 Euros	68 (18.2)
≥3000 Euros	31 (8.3)
Employed during pregnancy, *n* (%)	
No	108 (29.0)
Yes	265 (71.0)
Illness, *n* (%)	
No	326 (87.6)
Yes	46 (12.4)
Previous miscarriages, *n* (%)	
No	280 (75.1)
Yes	93 (24.9)
Planned pregnancy, *n* (%)	
No	47 (12.6)
Yes	326 (87.4)
Attended antenatal education, *n* (%)	
No	155 (41.6)
Yes	218 (58.4)
Pregnancy follow-up, *n* (%)	
Public system	182 (48.8)
Private system	7 (1.9)
In both	184 (49.3)
Fertility treatment, *n* (%)	
No	327 (87.7)
Yes	46 (12.3)
Gestation at birth, mean (SD)	39.43 (1.41)

* Baccalaureate (equivalent of A levels) /Professional formation. Abbreviations: n, number; SD, standard deviation.

**Table 2:** Association between maternal age and different parameters during pregnancy, labor, delivery and the puerperium.

Variable	Total, *n*	Crude Analysis		Multivariate Analysis	
		Age	*p*–Value	Age	a *p*–Value *
		Mean (95%CI)		Mean (95%CI)	
Health problems during pregnancy					
No	184	29.86 (29.08–30.64)	0.043	29.90 (29.13–30.68)	0.177
Yes	189	31.04 (30.20–31.87)		30.66 (29.89–31.43)	
Hypertension					
No	357	30.30 (29.72–30.88)	0.011	30.11 (29.56–30.66)	0.002
Yes	16	33.97 (30.61–37.33)		34.54 (31.80–37.27)	
Gestational diabetes					
No	345	29.98 (29.40–30.55)	<0.001	29.88 (29.34–30.42)	<0.001
Yes	28	36.35 (34.36–38.34)		36.01 (33.95–38.07)	
Anemia					
No	285	30.49 (29.82–31.15)	0.84	30.39 (29.76–31.01)	0.505
Yes	88	30.35 (29.20–31.51)		29.95 (28.82–31.07)	
TPTL					
No	358	30.55 (29.96–31.13)	0.116	30.35 (29.79–30.91)	0.234
Yes	15	28.21 (25.12–31.31)		28.68 (26.00–31.37)	
Emergency consult					
No	108	31.02 (30.04–32.00)	0.215	30.53 (29.51–31.56)	0.568
Yes	265	30.22 (29.52–30.93)		30.18 (29.52–30.83)	
Hospital Admission during pregnancy					
No	313	30.70 (30.08–31.31)	0.058	30.40 (29.81–31.00)	0.313
Yes	60	29.19 (27.60–30.78)		29.64 (28.27–31.01)	
Spontaneous onset of labor					
No	193	31.40 (30.63–32.17)	0.0008	31.35 (30.62–32.10)	<0.001
Yes	180	29.45 (28.61–30.28)		29.10 (28.33–29.88)	
Eutocic delivery					
No	151	32.19 (31.29–33.09)	<0.001	31.90 (31.05–32.74)	<0.001
Yes	220	29.24 (28.53–29.95)		29.17 (28.48–29.86)	
Cesarean section					
No	315	29.97 (29.36–30.58)	<0.001	29.90 (29.31–30.48)	0.001
Yes	58	33.09 (31.67–34.51)		32.46 (31.07–33.86)	
Preterm delivery					
No	361	30.39 (29.81–30.97)	0.253	30.22 (29.67–30.77)	0.21
Yes	12	32.29 (28.42–36.15)		32.27 (29.11–35.44)	
Medication during dilation					
No	41	29.34 (27.88–30.80)	0.18	28.94 (27.33–30.54)	0.08
Yes	332	30.59 (29.97–31.21)		30.46 (29.88–31.04)	
Epidural analgesia					
No	20	28.58 (26.19–30.98)	0.127	29.74 (27.37–32.10)	0.641
Yes	353	30.56 (29.97–31.15)		30.31 (29.75–30.87)	
Clear amniotic fluid					
No	54	31.90 (30.34–33.47)	0.039	32.42 (31.00–33.83)	0.001
Yes	318	30.19 (29.58–30.81)		29.89 (29.31–30.48)	
Non–reassuring cardiotocographic recording					
No	45	32.71 (31.07–34.36)	0.004	31.91 (30.37–33.46)	0.027
Yes	321	30.14 (29.52–30.76)		30.04 (29.45–30.63)	
Spontaneous third stage of labor					
No	131	31.92 (31.00–32.84)	<0.001	31.29 (30.35–32.23)	0.011
Yes	242	29.66 (28.95–30.38)		29.77 (29.11–30.44)	
Episiotomy					
No	81	31.65 (30.30–32.99)	0.444	31.29 (30.06–32.52)	0.563
Yes	138	31.02 (30.08–31.97)		30.83 (29.89–31.77)	
Perineal tear					
No	80	31.65 (30.30–32.99)	0.002	31.20 (30.01–32.38)	0.005
Yes	153	29.24 (28.41–30.08)		29.09 (28.25–29.92)	
Postpartum complications					
No	337	30.29 (29.69–30.88)	0.079	30.08 (29.51–30.65)	0.029
Yes	36	32.02 (29.94–34.11)		32.15 (30.39–33.92)	

* Adjusted for education level, income level, maternal smoking habit, history of previous miscarriage and presence of medical pathology prior to pregnancy. Abbreviations: CI, confidence interval; n, number; TPTL, threatened preterm labor.

**Table 3:** Analysis of pregnancy, birth and puerperium variables stratified for age.

Variable	Total, *n*	Age (years)			
		<25 *n* (%)	25–29 *n* (%)	30–34 *n* (%)	≥35 *n* (%)
Health problems during pregnancy					
No	184	31(60.78)	39 (44.32)	71 (52.99)	43 (43.00)
Yes	189	20(39.22)	49 (55.68)	63 (47.01)	57 (57.00)
OR (95%CI)		1 ref.	1.94 (0.96–3.93)	1.38 (0.71–2.65) 0.341	2.05 (1.03–4.09) 0.040
*p*			0.063		
a OR * (95%CI),		1 ref.	2.04 (0.96–4.35) 0.064	1.42 (0.67–2.97) 0.357	1.79 (0.81–3.98)
a *p*–Value *					0.152
Hypertension					
No	357	No cases	136 (97.84)	129 (96.27)	92 (92.00)
Yes	16		3 (2.16)	5 (3.73)	8 (8.00)
OR (95%CI)			1 ref.	1.16 (0.22–6.17) 0.859	2.61 (0.54–12.71) 0.235
*p*					
a OR * (95%CI),			1 ref.	3.66 (0.65–20.59) 0.141	7.34 (1.32–40.91)
a *p*–Value *					0.023
Gestational diabetes					
No	345	No cases	137 (98.56)	126 (94.03)	82 (82.00)
Yes	28		2 (1.44)	8 (5.97)	18 (18.00)
OR (95%CI),			1 ref.	4.35 (0.91–20.87)	15.04 (3.40–66.47)
*p*					
a OR * (95%CI),			1 ref.	5.23 (0.99–27.57) 0.051	15.18 (3.03–76.00) 0.001
a *p*–Value *					
Anemia					
No	285	40(78.43)	65 (77.86)	98 (73.13)	82 (82.00)
Yes	88	11(21.57)	23 (26.14)	36 (26.87)	18 (18.00)
OR (95%CI),		1 ref.	1.29 (0.57–2.92) 0.547	1.34 (0.62–2.88) 0.460	0.80 (0.34–1.85) 0.599
*p*					
a OR * (95%CI),		1 ref.	1.29 (0.54–3.12) 0.563	1.22 (0.52–2.93)	0.62 (0.23–1.67) 0.349
a *p*–Value *				0.641	
TPTL					
No	358	47 (92.16)	83 (94.32)	132 (98.51)	96 (96.00)
Yes	15	4 (7.84)	5 (5.68)	2 (1.49)	4 (4.00)
OR (95%CI)		1 ref.	0.71 (0.18–2.77) 0.619	0.18 (0.03–1.00)	0.49 (0.11–2.04) 0.327
*p*				0.051	
a OR * (95%CI),		1 ref.	0.72 (0.17–3.02)	0.21 (0.03–1.33)	0.56 (0.98–3.17)
a *p*–Value *			0.656	0.098	0.51
Emergency room visit					
No	108	12 (23.53)	20 (22.73)	47 (35.07)	29 (29.00)
Yes	265	39 (76.47)	68 (77.27)	87 (64.93)	71 (71.00)
OR (95%CI)		1 ref.	0.96 (0.43–2.12) 0.912	0.24 (0.27–1.08) 0.081	0.71 (0.34–1.49) 0.370
*p*					
a OR * (95%CI),		1 ref.	1.00 (0.42–2.41) 0.996	0.55 (0.25–1.24) 0.150	0.82 (0.34–1.97) 0.653
a *p*–Value *					
Hospital admission during pregnancy					
No	313	40 (78.43)	69 (78.43)	119 (88.81)	85 (85.00)
Yes	60	11 (21.57)	19 (29.59)	15 (11.19)	15 (15.00)
OR (95%CI)		1 ref.	0.64 (0.28–1.44) 0.279	0.36 (0.17–0.79) 0.011	0.51 (0.23–1.12)
*p*					0.092
a OR * (95%CI),		1 ref.	0.72 (0.30–1.74) 0.462	0.45 (0.18–1.09) 0.076	0.66 (0.26–1.68) 0.379
a *p*–Value *					
Spontaneous onset of labor					
No	193	16 (31.37)	41 (46.59)	76 (56.72)	60 (60.00)
Yes	180	35 (68.63)	47 (53.41)	58 (43.28)	40 (40.00)
OR (95%CI)		1 ref.	1.91 (0.92–3.94) 0.081	2.87 (1.45–5.68) 0.003	3.28 (1.61–6.70) 0.001
*p*					
a OR * (95%CI),		1 ref.	2.69 (1.21–5.99) 0.016	4.45 (2.00–9.89) <0.001	5.87 (2.47–13.96) <0.001
a *p*–Value *					
Eutocic delivery					
No	151	11 (21.57)	30 (64.48)	53 (39.55)	57 (57.58)
Yes	220	40 (78.43)	57 (65.52)	81 (60.45)	42 (42.42)
OR (95%CI)		1 ref.	1.91 (0.86–4.23) 0.112	2.38 (1.12–5.05) 0.024	4.94 (2.27–10.74) <0.001
*p*					
a OR * (95%CI),		1 ref.	2.52 (1.05–6.06) 0.038	3.71 (1.56–8.80) 0.003	6.92 (2.74–17.48) <0.001
a *p*–Value *					
Cesarean section					
No	315	50 (98.04)	76 (86.36)	112 (83.58)	77 (77.00)
Yes	58	1 (1.96)	12 (13.64)	22 (16.42)	23(23.00)
OR (95%CI)		1 ref.	7.89 (1.00–62.63) 0.051	9.82 (1.29–74.90) 0.028	14.93 (1.95–114.12)
*p*					0.009
a OR * (95%CI),		1 ref.	9.81 (1.21–79.84)	13.29 (1.64–107.64)	16.32 (1.97–135.26)
a *p*–Value *				0.015	0.01
Preterm delivery					
No	361	50 (98.04)	86 (97.73)	130 (97.01)	95 (95.00)
Yes	12	1 (1.96)	2 (2.27)	4 (2.99)	5 (5.00)
OR (95%CI)		1 ref.	1.16 (0.10–13.15) 0.903	1.54 (0.17–14.10) 0.703	2.63 (0.30–23.14) 0.383
*p*					
a OR * (95%CI),		1 ref.	1.38 (0.12–16.42) 0.796	1.87 (0.16–21.82), 0.618	3.48 (0.31–39.06) 0.312
a *p*–Value *					
Medication during dilation					
No	41	6 (11.76)	11 (12.50)	19 (14.18)	5 (5.00)
Yes	332	45(88.24)	77 (87.50)	115 (85.82)	95 (95.00)
OR (95%CI)		1 ref.	0.93 (0.32–2.70) 0.899	0.81 (0.30–2.15) 0.668	2.53 (0.73–8.74) 0.141
*p*					
a OR * (95%CI),		1 ref.	1.01 (0.33–3.09) 0.984	1.07 (0.36–3.15) 0.904	3.68 (0.88–15.40) 0.074
a *p*–Value *					
Epidural analgesia					
No	20	5 (9.80)	6 (6.82)	5 (3.73)	4 (4.00)
Yes	353	46 (90.20)	82 (93.18)	129 (96.27)	96 (96.00)
OR (95%CI)		1 ref.	1.49 (0.43–5.14) 0.532	2.80 (0.78–10.13) 0.116	2.61 (0.67–10.17) 0.167
*p*					
a OR * (95%CI),		1 ref.	0.80 (0.18–3.55) 0.770	1.51 (0.31–7.28) 0.605	1.10 (0.20–5.99) 0.909
a *p*–Value *					
Clear amniotic fluid					
No	54	6 (11.76)	10 (11.36)	17 (12.69)	21 (21.21)
Yes	318	45 (88.24)	78 (88.64)	117 (87.31)	78 (78.79)
OR (95%CI)		1 ref.	0.96 (0.33–2.82) 0.943	1.09 (0.40–2.94) 0.865	2.02 (0.76–5.37) 0.159
*p*					
a OR * (95%CI),		1 ref.	1.56 (0.49–5.02) 0.455	2.10 (0.67–6.60) 0.203	4.61 (1.42–14.99) 0.011
a *p*–Value *					
Non–reassuring cardiotocographic recording					
No	45	3 (5.88)	8 (9.52)	14 (10.61)	20 (20.20)
Yes	321	48 (94.12)	76 (90.48)	118 (89.39)	79 (79.80)
OR, (95%CI)		1 ref.	1.68 (0.43–6.66) 0.458	1.90 (0.52–6.91) 0.331	4.05 (1.14–14.36) 0.030
*p*					
a OR * (95%CI),		1 ref.	1.69 (0.41–6.86) 0.465	1.84 (0.48–7.10) 0.377	3.62 (0.92–14.23) 0.066
a *p*–Value *					
Spontaneous third stage of labor					
No	131	7 (13.73)	32 (36.36)	51 (38.06)	41 (41.00)
Yes	242	44 (86.27)	56 (63.64)	83 (61.94)	59 (59.00)
OR (95%CI)		1 ref.	3.59 (1.45–8.91) 0.006	3.86 (1.62–9.22) 0.002	4.37 (1.79–10.65) 0.001
*p*					
a OR * (95%CI),		1 ref.	4.21 (1.56–11.35) 0.005	4.19 (1.56–11.22) 0.004	3.82 (1.36–10.73) 0.011
a *p*–Value *					
Episiotomy					
Intact perineum	80	7 (31.82)	18 (38.30)	30 (37.04)	25 (36.76)
Yes	138	15 (68.18)	29 (61.70)	51 (62.96)	43 (63.24)
OR (95%CI)		1 ref.	0.75 (0.26–2.20) 0.602	0.79 (0.29–2.17) 0.651	0.80 (0.29–2.23) 0.674
*p*					
a OR * (95%CI),		1 ref.	0.69 (0.22–2.17) 0.526	0.74 (0.24–2.31) 0.599	0.79 (0.24–2.63) 0.707
a *p*–Value *					
Perineal tear					
Intact perineum	80	7 (19.44)	18 (30.51)	30 (36.14)	25 (45.45)
Yes	153	29 (80.56)	41 (69.49)	53 (63.86)	30 (54.55)
OR (95%CI)		1 ref.	0.55 (0.20–1.49) 0.238	0.43 (0.17–1.09) 0.075	0.29 (0.11–0.77) 0.013
*p*					
a OR * (95%CI),		1 ref.	0.42 (0.14–1.22) 0.112	0.27 (0.09–0.79) 0.017	0.22 (0.07–0.71) 0.011
a *p*–Value *					
Postpartum complications					
No	353	49 (96.08)	82 (93.18)	128 (95.52)	94 (94.0)
Yes	20	2 (3.92)	6 (6.82)	6 (4.48)	6(6.0)
OR (95%CI)		1 ref.	1.79 (0.35–9.23) 0.485	1.15 (0.22–5.88) 0.868	1.56 (0.30–8.04) 0.592
*p*					
a OR * (95%CI),		1 ref.	1.92 (0.36–10.33) 0.446	0.65 (0.97–4.30) 0.651	2.20 (0.37–12.95) 0.384
a *p*–Value *					

* Adjusted for education level, income level, maternal smoking habit, previous miscarriages and presence of any pathology prior to pregnancy. Abbreviations: CI, confidence interval, OR, odds ratio; TPTL, threatened preterm labor.

**Table 4:** Association between maternal age and different continuous variables during pregnancy, labor and the puerperium.

Variable	Total, Mean, SD	Crude Analysis					Multivariate Analysis				
		Age (years)				*p*-Value	Age (years)				a *p*-Value *
		<25	25–29	30–34	≥35		<25	25–29	30–34	≥35	
Number of visits to the Emergency department	1.5	1.96	1.47	1.24	1.66	0.064	1.95	1.46	1.28	1.67	0.087
	±1.53	± 0.27	±0.13	±0.11	±0.18		±0.24	±0.17	±0.14	±0.17	
Number of inpatient days following delivery	2.41	2.25	2.58	2.28	2.54	0.175	2.28	2.62	2.28	2.51	0.165
	±1.25	±0.13	±0.26	±0.06	±0.11		±0.20	±0.15	±0.12	±0.15	
Duration of dilatation	356.68	299.51	363.73	347.79	392.3	0.024	290.92	365.65	356.38	398.68	0.028
(min.)	±180.45	±22.93	±19.91	±15.50	±18.78		±27.87	±20.55	±16.43	±19.89	
Duration of second stage labor	99.9	100.6	99.75	95.84	105.5	0.732	109.6	102.36	91.33	101.39	0.537
(min.)	±59.81	±8.11	±6.35	±13.04	±7.18		±9.49	±7.18	±5.78	±7.24	
Duration of third stage of labor (min.)	9.17	9.82	9.96	8.66	8.8	0.557	10.81	10.41	8.45	8.6	0.789
	±7.63	±0.92	±1.05	±0.58	±0.77		±1.22	±0.88	±0.71	±0.87	
Pain during labor	6.76	6.57	6.6	6.79	6.97	0.676	6.44	6.61	6.76	6.87	0.706
(0–10)	±2.30	±0.34	±0.26	±0.19	±0.23		±0.38	±0.27	±0.22	±0.27	
Gestational week of delivery	39.43	39.39	39.67	39.38	39.29	0.295	39.36	39.66	39.43	39.31	0.254
	±1.42	±0.17	±0.14	±0.12	±0.16		±0.22	±0.16	±0.13	±0.16	

* Adjusted for education level, income level, maternal smoking habit, previous history of miscarriage and previous medical history prior to pregnancy. Abbreviation: SD, standard deviation.
